# Predictors of COVID-19 vaccine hesitancy in the UK household longitudinal study

**DOI:** 10.1016/j.bbi.2021.03.008

**Published:** 2021-05

**Authors:** Elaine Robertson, Kelly S. Reeve, Claire L. Niedzwiedz, Jamie Moore, Margaret Blake, Michael Green, Srinivasa Vittal Katikireddi, Michaela J. Benzeval

**Affiliations:** a MRC/CSO Social & Public Health Sciences Unit, University of Glasgow, UK; b Understanding Society, Institute for Social and Economic Research, University of Essex, UK; c Institute of Health & Wellbeing, College of Medical, Veterinary and Life Sciences, University of Glasgow, UK; d Ipsos MORI UK Ltd, UK; e Public Health Scotland, UK

**Keywords:** COVID-19, Vaccine hesitancy, Vaccine uptake, Ethnicity, Socioeconomic position, Inequalities

## Abstract

Vaccine hesitancy could undermine efforts to control COVID-19. We investigated the prevalence of COVID-19 vaccine hesitancy in the UK and identified vaccine hesitant subgroups. The ‘Understanding Society’ COVID-19 survey asked participants (n = 12,035) their likelihood of vaccine uptake and reason for hesitancy. Cross-sectional analysis assessed vaccine hesitancy prevalence and logistic regression calculated odds ratios. Overall vaccine hesitancy was low (18% unlikely/very unlikely). Vaccine hesitancy was higher in women (21.0% vs 14.7%), younger age groups (26.5% in 16–24 year olds vs 4.5% in 75 + ) and those with lower education levels (18.6% no qualifications vs 13.2% degree qualified). Vaccine hesitancy was high in Black (71.8%) and Pakistani/Bangladeshi (42.3%) ethnic groups. Odds ratios for vaccine hesitancy were 13.42 (95% CI:6.86, 26.24) in Black and 2.54 (95% CI:1.19, 5.44) in Pakistani/Bangladeshi groups (compared to White British/Irish) and 3.54 (95% CI:2.06, 6.09) for people with no qualifications versus degree. Urgent action to address hesitancy is needed for some but not all ethnic minority groups.

## Highlights

• Willingness to be vaccinated was generally high across the UK population.
• Vaccine hesitancy exists in population subgroups.
• Black and Pakistani/Bangladeshi ethnic groups had higher levels of vaccine hesitancy.
• Vaccine hesitancy levels are higher in people with lower education levels.
• Focus on ethnic minority and socioeconomic groups is needed during vaccine delivery.

## 1 Introduction

Vaccines and immunisation programmes save lives. The World Health Organisation (WHO) estimates that immunisation programmes across the world prevent 2–3 million deaths every year and are not only cost effective but a key element of preventative healthcare (WHO, 2020). Since the emergence of the Severe Acute Respiratory Syndrome Coronavirus 2 (SARS-CoV-2) in 2019, the global pandemic has resulted in over 72 million confirmed cases and 1.6 million deaths in 220 countries worldwide (as at 18 Dec 2020) (WHO, 2020). An effective and safe vaccine is vital to controlling the COVID-19 outbreak. However, not only does a vaccine need to be safe and effective, it must also be taken up by those people at greatest risk of harm from the disease. Ideally, uptake by a large enough proportion of the population will offer protection to people who remain unimmunised, referred to as achieving ‘herd immunity’. For COVID-19, vaccine uptake would need to be between approximately 67% and 80% to reduce spread of the disease (Randolph and Barreiro, 2020, Mills et al., 2020). Understanding who will take up a vaccine, who plans not to or are uncertain, and why, is critical to designing a successful vaccination programme.

Even before the emergence of SARS-CoV-2, WHO had already highlighted vaccine hesitancy as one of the ten leading threats to global health (WHO, 2019). The WHO Strategic Advisory Group of Experts Working Group on Vaccine Hesitancy define it as the “*delay in acceptance or refusal of safe vaccines despite availability of vaccination services*” (MacDonald, 2015). Reasons for vaccine hesitancy are acknowledged as being complex but also context specific. Hesitancy around uptake can vary geographically, at different times and for different vaccines by a range of factors including complacency around the disease, convenience of access and confidence in the vaccine itself. For example, in the H1N1 pandemic of 2009, there was a perception that the vaccine was rushed and unsafe (Schoch-Spana et al., 2020). Similar concerns have been raised about the speed of COVID-19 vaccine development (Mills et al., 2020). Studies into potential COVID-19 vaccination, based on non-representative samples, have indicated that vaccine hesitancy associated with age, gender, income, education level and ethnicity will be an issue (Ipsos, 2020, Murphy et al., 2021, Paul et al., 2021, Williams et al., 2021). In studies conducted prior to vaccine approval, hesitancy rates for a COVID-19 vaccine in the general UK population varied greatly, from approximately 12% (Freeman et al., 2020) to 31%. (Murphy et al., 2021) In studies where ethnicity was considered, small sample sizes did not allow for detailed analysis of specific ethnic groups, but suggested greater hesitancy amongst people classified as non-White (Murphy et al., 2021, Paul et al., 2021, Williams et al., 2021, Freeman et al., 2020). This is particularly important, given the often greater risk of COVID-19 harms observed among several ethnic minority groups (Lassale et al., 2020, Niedzwiedz et al., 2020, Williamson et al., 2020).

The COVID-19 pandemic has been described as a ‘syndemic’ where it not only acts together with, but worsens and amplifies, non-communicable diseases and social conditions and hence exacerbates inequalities in health (Bambra et al., 2020). As inequalities already exist in seasonal influenza and pneumococcal vaccine uptake, with lower uptake in more deprived areas (Mangtani et al., 2005, Jain et al., 2017), there is reason to believe that inequalities will exist in vaccine uptake against COVID-19. Understanding if some subgroups of the population are more likely to be vaccine hesitant will help in the formulation of vaccination programme strategies to ensure adequate population coverage to achieve herd immunity and minimise health inequalities. This will be particularly important if greater vaccine hesitancy is found among groups at greater risk from COVID-19. We therefore aimed to describe how willing the UK population is to be vaccinated against COVID-19, to identify which population subgroups are more likely to be vaccine hesitant, which are more likely to take up a vaccine, and describe the main reasons given for both vaccine uptake and vaccine hesitancy.

## 2 Methods

### 2.1 Data

The UK Household Longitudinal Study, also referred to as ‘Understanding Society’, is a nationally representative panel study, based on a clustered-stratified probability sample of UK households, with boost samples of key ethnic minority groups. Sample members living in the UK have been interviewed annually since 2009 (Institute for Social and Economic Research, 2020). In 2020, during the COVID-19 pandemic, participants aged 16+ years who had lived in households that had completed at least one of the last two waves of the main Understanding Society survey were invited to take part in the COVID-19 survey either online or by telephone (n = 42,330). Web surveys took place monthly from April to July, then every two months (Institute for Social and Economic Research, 2020). For wave 6, carried out from 24th November to 1st December 2020, only sample members who had completed at least one partial interview in any of the preceding five COVID-19 web surveys, and had not become ineligible through death or moving abroad nor opted out of the study, were invited to take part. This resulted in 19,289 invitations being issued, and 12,035 took part, a response rate of 62% (University of Essex, 2020). Data for age, sex, ethnicity, education level and country of birth were derived from previous waves of the main study (Institute for Social and Economic Research, 2020).

Ethics approval was granted by the University of Essex Ethics Committee for the COVID-19 surveys (ETH1920-1271). No additional ethical approval was necessary for this secondary data analysis.

### 2.2 Outcome measures

Our primary outcome was vaccine hesitancy, assessed by asking all participants “Imagine that a vaccine against COVID-19 was available for anyone who wanted it. How likely or unlikely would you be to take the vaccine?” Possible responses were “Very likely”, “Likely”, “Unlikely” and “Very unlikely”. This was collapsed into a binary variable for modelling, comparing ‘unlikely and very unlikely’, classified as vaccine hesitant, to ‘likely or very likely’. If a participant tried to bypass the question, they were given the option of ‘don’t know’ (n = 45) and, given the size of the group they were coded as missing.

Secondary outcomes included reasons for vaccine hesitancy. Vaccine hesitant participants were asked “What is the main reason you would not take the vaccine?” and asked to pick one main reason from a list of 12 possible answers (Fig. 3). Participants who were not vaccine hesitant were asked *“What would be your main reason for taking the vaccine?”* and asked to choose one main reason from a list of 11 possible answers (Fig. 3). All participants were then asked “*Which three of these things would most increase the chances of you choosing to get vaccinated?*” and given a list of 9 possible answers (Fig. 3). For the purpose of this analysis, we identified if the item was mentioned as any of the three options.

### 2.3 Covariates

Covariates are all taken from the longitudinal mainstage data files. Statistical models included age, coded in 5-year age bands to allow for non-linearity in the relationship, and gender coded as male/female. Ethnicity was self-reported and coded as White British or Irish; Other White; three Asian and Asian British groups: Indian, Pakistani/Bangladeshi, or Other Asian (includes participants of Chinese ethnicity); Black or Black British; and Other. The ethnicity groupings were chosen to allow analysis of as detailed ethnic groupings as possible, subject to having an adequate sample size for meaningful analysis in each category. Country of birth (UK/Not UK) and UK country of residence (England/Scotland/Wales/Northern Ireland) were also investigated. Education level was based on the highest qualification reported in the most recent wave (data collected in 2019) and coded as Degree & Other Higher Degree, A-Level or equivalent, GCSE or equivalent, other qualification and none. NHS Shielding category (Yes/No) was ascertained from previous COVID-19 survey waves on the basis of self-report (“*Have you received a letter, text or email from the NHS or Chief Medical Officer saying that you have been identified as someone at risk of severe illness if you catch coronavirus, because you have an underlying disease or health condition?*”).

### 2.4 Statistical analysis

Descriptive statistics (percentages and 95% confidence intervals (CI)) for the outcomes were calculated using cross-sectional weights to make the sample representative of the UK community dwelling population. Weights were calculated for each COVID-web survey based on differential non-response from wave 9. Predictors included in the weights were basic demographic factors, household composition, previous survey outcomes, COVID-19 survey paradata, such as the number of reminders issued, economic and health variables. The final weights are calculated as the inverse of the response propensity. (Institute for Social and Economic Research, 2020) Weights are not calculated for the 1,974 participants who did not take part at Wave 9, and hence they are excluded from the weighted analyses, hence the weighted sample is 9,981.

Logistic regression was used because the dependent variable was binary. Three models were considered: model 0 - univariate models for each covariate, model 1 – statistical analysis including age and gender, model 2 – statistical analysis including all covariates.

Data were cleaned using SPSS version 25 and analysed in R version 3.6.3 (R Core Team, 2018) using the complex samples method to take account of the clustered and stratified sample (Lumley, 2020). Missing data were excluded from the analyses using listwise deletion so each model may contain different numbers of participants.

Data Availability

Understanding Society data are available through the UK Data Service. COVID-19 Survey is available here https://beta.ukdataservice.ac.uk/datacatalogue/studies/study?id=8644, and the mainstage here https://beta.ukdataservice.ac.uk/datacatalogue/studies/study?id=6614. Researchers who would like to use Understanding Society need to register with the UK Data Service before being allowed to apply for or download datasets.

Code availability

This project has employed statistical analytical techniques standard in all statistical packages.

## 3 Results

### 3.1 Sample statistics

**Fig. 1:**
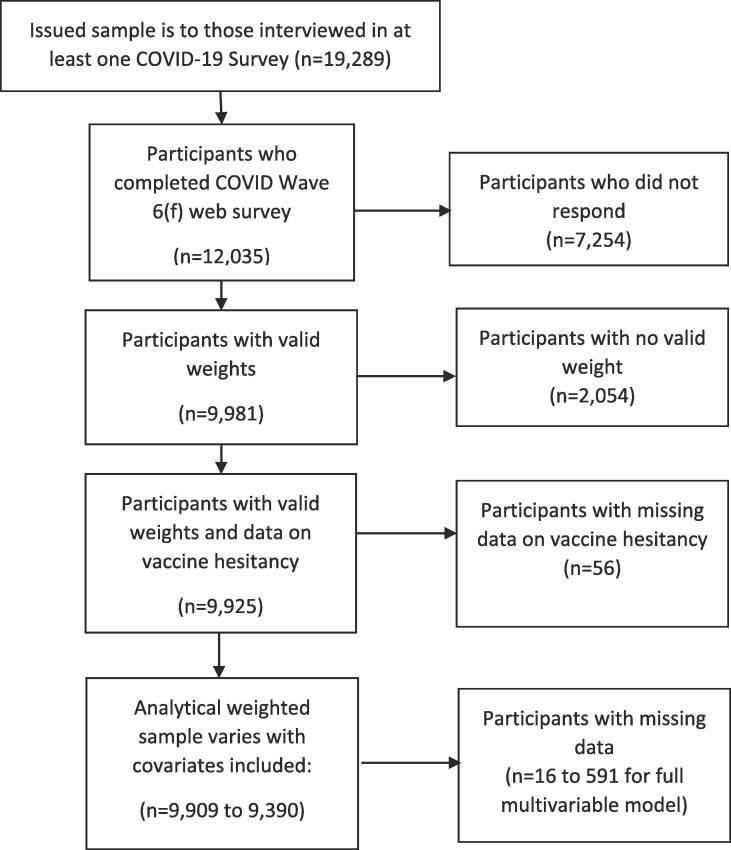
Selection of analytical sample.

12,035 participants completed the Covid-19 wave 6 survey online and the weighted sample is 9,981 (Fig. 1). However, in the weighted sample 56 participants did not answer the vaccine hesitancy question and 591 participants had missing data on at least one covariate, and hence the weighted analytical sample for the full multivariable models is 9,390, although in other models it varies based on the covariates included. The weighted study sample is described in Table 1.

**Table 1:** Description of the study population.

	Weighted n (%)
Likelihood of taking up a coronavirus vaccination	
Very likely	5306 (53.2%)
Likely	2832 (28.4%)
Unlikely	1131 (11.3%)
Very unlikely	656(6.6%)
Missing (including refusal and don’t know)	56 (0.5%)
Gender	
Male	4666 (46.8%)
Female	5290 (53.0%)
Prefer not to say	25 (0.3%)
Age	
16–24	920 (9.2%)
25–34	1382(13.8%)
35–44	1543 (15.5%)
45–54	1784 (17.9%)
55–64	1938 (19.4%)
65–74	1532 (15.3%)
75+	882 (8.8%)
Ethnicity	
White British or Irish	8713 (87.3%)
Other white background	269 (2.7%)
Mixed	168 (1.7%)
Asian or Asian British – Indian	176 (1.8%)
Asian or Asian British – Pakistani/Bangladeshi	198 (2.0%)
Asian or Asian British – any other group	106 (1.1%)
Black or Black British	190 (1.9%)
Other Ethnic Group	59 (0.6%)
Missing	102 (1.0%)
Born in UK	
Born in UK	8991 (90.1%)
Not Born in UK	824 (8.3%)
Missing	166 (1.7%)
UK Country	
England	8424 (84.4%)
Wales	507 (5.1%)
Scotland	775 (7.8%)
Northern Ireland	275 (2.8%)
Highest Education Level	
Degree & Other Degree	4086 (40.9%)
A Level or equivalent	2202 (22.1%)
GCSE or equivalent	2010 (20.1%)
Other Qualification	846 (8.5%)
No Qualification	501 (5.0%)
Missing(no education data in 2019 datafile (wave 10/11)	336 (3.4%)
NHS Shielding Category	
Yes	721 (7.2%)
No	9244 (92.6%)
Don’t know	16 (0.2%)

Unweighted N = 11,955, weighted N = 9,981.

### 3.2 Prevalence of vaccine hesitancy

**Fig. 2:**
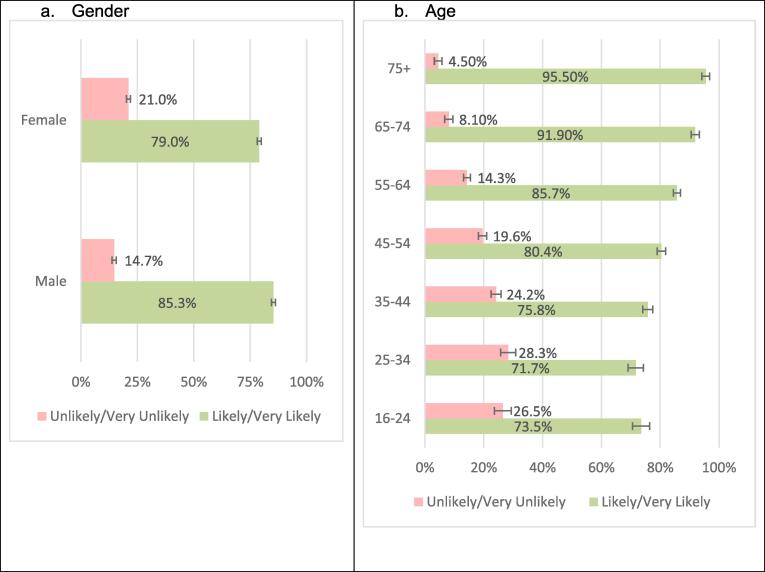

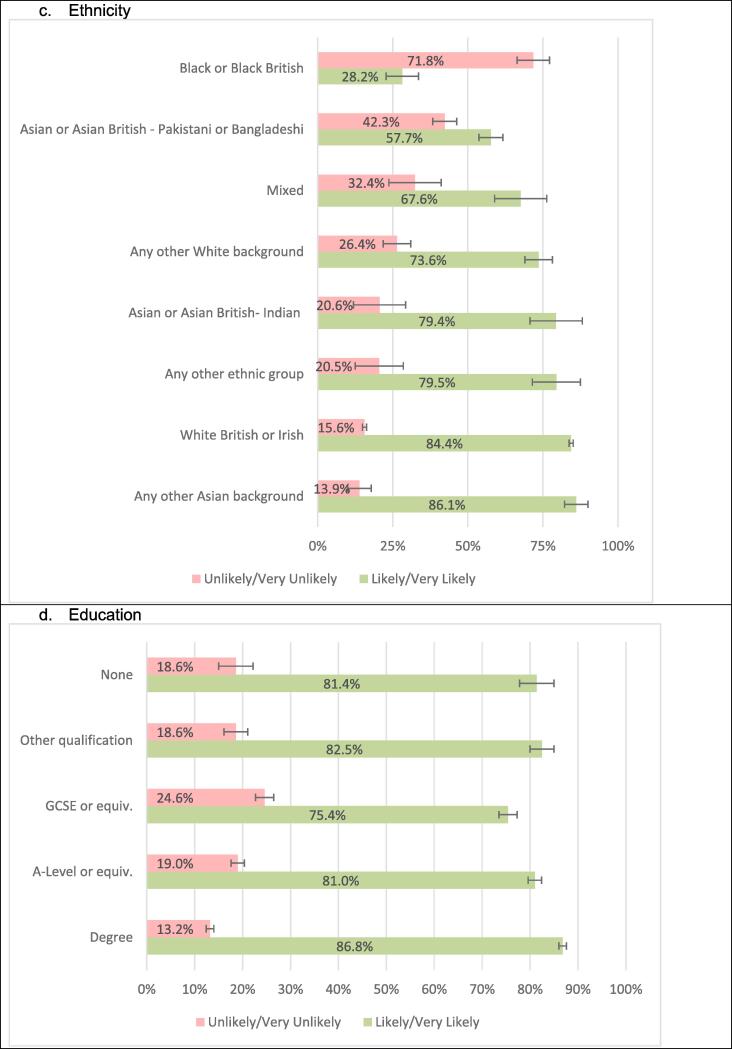
Proportions of vaccine hesitancy and willingness to be vaccinated (weighted proportions with 95% CI).

Overall, intention to have the COVID-19 vaccine was high, 53.5% of participants were very likely and a further 28.5% were likely to be vaccinated with 18% being vaccine hesitant (reporting unlikely or very unlikely). However, there was marked variation in population subgroups (Fig. 2). A higher proportion of female participants indicated vaccine hesitancy, 21.0% compared to 14.7% of male participants. Younger age groups were also more vaccine hesitant with 28.3% of younger adults aged 25–34 vaccine hesitant compared to only 14.3% in the 55–64 age group, 8.1% in the 65–74 age group and 4.5% in the 75 + age group.

**Fig. 3:**
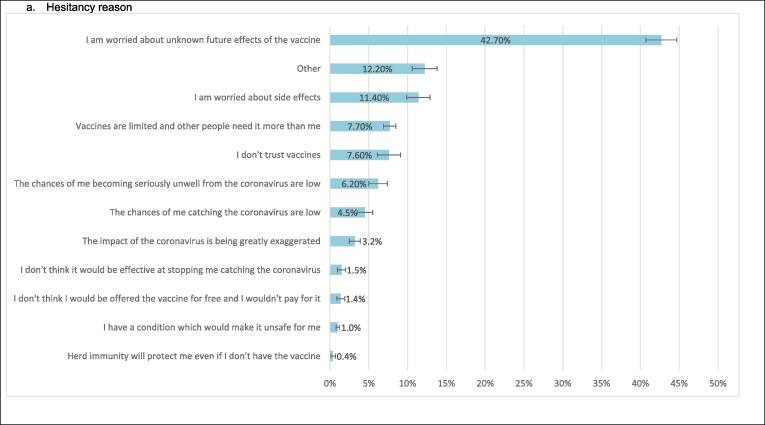

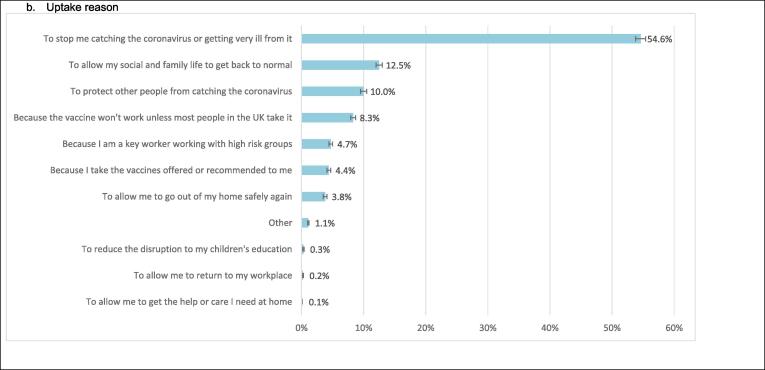

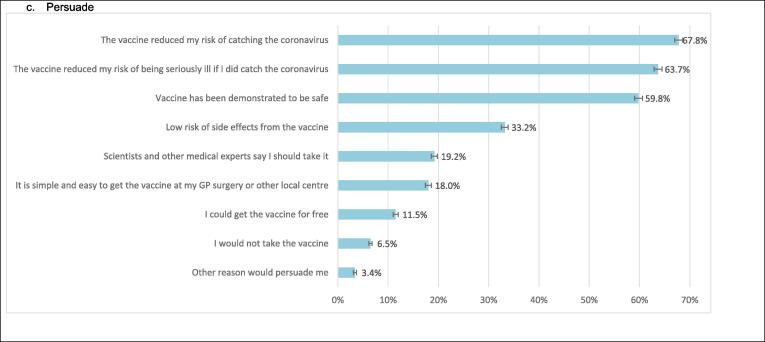
Reasons for vaccine hesitancy, willingness to take vaccines and factors that would persuade people to take a vaccine (weighted proportions with 95% CI bars).

Black or Black British were the ethnic group with the highest rate of vaccine hesitancy at 71.8%. Pakistani/Bangladeshi groups were the next most hesitant ethnic group with 42.3% vaccine hesitant, followed by those of Mixed ethnicity (32.4%). The ethnic groups with the highest intention to vaccinate were the White British or Irish groups (84.8% being likely/very likely to take a vaccine) and the any other Asian background group (86.1%; this group includes participants of Chinese ethnicity).

Vaccine hesitancy varied with education level. Vaccine hesitancy was lowest in those with degrees (13.2%) and highest in those with GCSE level education (24.6%).

### 3.3 Reasons for vaccine hesitancy

The main reasons for vaccine hesitancy were concerns over future unknown effects of a vaccine, with 42.7% citing this as their main reason (Fig. 3). The main reasons for being willing to take up a vaccine were to avoid catching COVID-19 or becoming ill from the disease (54.6%) and to allow social and family life to get back to normal (12.5%).

Stated reasons for vaccine hesitancy were often similar across ethnic groups (Appendix 1). However, when compared to the White British or Irish group, Black or Black British participants were more likely to state they ‘Don’t trust vaccines’ (29.2% vs 5.7%) and the Pakistani or Bangladeshi ethnic group cited worries about side-effects (35.4% vs 8.6%) more commonly. Women were more likely than men to state that their main reason for vaccine hesitancy was concern about side effects and to state that they do not trust vaccines.

A majority of participants (67.8%) reported that knowing a vaccine reduced their risk of catching COVID-19 was a factor that would increase their chance of taking a vaccine (Fig. 3). Other factors commonly identified by participants as increasing their chances of taking a vaccine were if it reduced the risk of being seriously ill if they did catch COVID-19 (63.7%) and a vaccine being demonstrated to be safe (59.8%).

When asked what would most convince participants to take the vaccine, 43.2% of Black or Black British maintained that they would not take it, while a further 44.7% reported that they would if the vaccine was demonstrated to be safe. Pakistani/Bangladeshi participants reported that they may be persuaded if the vaccine reduced their risk of catching COVID-19 (65.2%) and/or if it was demonstrated to be safe (64.6%).

Females had higher odds of vaccine hesitancy than males (OR 1.55, 95% CI:1.28, 1.86) and this was still present in model 1 (OR 1.54, 95% CI:1.28, 1.86) (Table 2). The risk of being vaccine hesitant was inversely related to age with younger age groups having higher odds of vaccine hesitancy; the odds in the 16–24 year old category were 1.48 (95% CI:1.00, 2.20) compared to those aged 45–54 years. However, once adjusting for other covariates this was reduced to 1.29 (95% CI: 0.85, 1.95). The vaccine hesitancy risk was higher in the 25–34 (OR 1.64, 95% CI: 1.16, 2.32) and 35–44 (OR 1.42, 95% CI:1.06, 1.89) than 45–54 year olds, and this was present in all models. Conversely those in older age groups were less likely to be vaccine hesitant across all models, 55–64 (OR 0.61, (95% CI:0.46, 0.80), 65–74 (OR 0.33, 95% CI:0.23, 0.46) and 75+ (OR 0.17, 95% CI:0.08, 0.35). For those participants who had received a letter about shielding, the odds of vaccine hesitancy were not different to those who are not shielding but were imprecisely estimated, and this was true across all models (OR 1.08, 95% CI:0.67, 1.74).

**Table 2:** Logistic regression models for vaccine hesitancy.

	**Model 0 Unadjusted Regression**		**Model 1 Age & Gender**			**Model 2 All covariates^1^**	
	**OR**	**95% CI**	**OR**	**95% CI**	**Predicted Probability^2^**	**OR**	**95% CI**
**Gender**							
Male (Ref)	1	–	1	–	15.9%	1	–
Female	1.546	1.283, 1.863	1.539	1.275, 1.858	22.6%	1.674	1.392, 2.015
**Age**							
16–24	1.479	0.995, 2.198	1.503	1.021, 2.213	22.0%	1.286	0.849, 1.948
25–34	1.624	1.181, 2.232	1.669	1.210, 2.301	23.9%	1.643	1.162, 2.323
35–44	1.309	0.991, 1.729	1.313	0.994, 1.735	19.8%	1.419	1.064, 1.892
45–54 (Ref)	1	–	1	–	15.8%	1	–
55–64	0.688	0.521, 0.907	0.692	0.525, 0.912	11.5%	0.608	0.464, 0.796
65–74	0.362	0.239, 0.547	0.366	0.243, 0.553	6.4%	0.327	0.231, 0.464
75+	0.194	0.102, 0.370	0.197	0.104, 0.375	3.6%	0.171	0.084, 0.348
**Ethnicity**							
White British or Irish (Ref)	1	–	1	–	10.8%	1	–
Other white background	1.935	1.193, 3.140	1.758	1.097, 2.818	17.4%	1.651	0.963, 2.829
Mixed	2.590	1.088, 6.168	1.735	0.801, 3.757	11.9%	2.091	0.972, 4.501
Asian or Asian British – Indian	1.383	0.805, 2.374	1.113	0.637, 1.946	26.0%	1.175	0.617, 2.238
Asian or Asian British – Pakistani/Bangladeshi	3.962	2.047, 7.668	2.906	1.502, 5.623	7.5%	2.544	1.189, 5.444
Asian or Asian British – any other group	0.873	0.323, 2.359	0.667	0.238, 1.866	64.6%	0.852	0.306, 2.377
Black or Black British	13.530	7.569, 24.185	15.034	7.619, 29.665	12.5%	13.417	6.862, 26.236
Other Ethnic Group	1.399	0.499, 3.923	1.180	0.385, 3.611	17.6%	1.407	0.495, 4.004
**Birth Country**							
Born in UK (Ref)	1	–	1	–	15.0%	1	–
Not Born in UK	1.952	1.391, 2.739	1.733	1.193, 2.518	23.4%	0.994	0.667, 1.482
**Shielding**							
No (Ref)	1	–	1	–	16.0%	1	–
Yes	0.780	0.486, 1.253	1.146	0.711, 1.846	17.9%	1.080	0.669, 1.744
**UK Country of residence**							
England (Ref)	1	–	1	–	16.4%	1	–
Wales	1.140	0.694, 1.871	1.105	0.674, 1.811	17.8%	1.121	0.692, 1.818
Scotland	0.603	0.438, 0.831	0.612	0.441, 0.849	10.7%	0.817	0.583, 1.143
N Ireland	1.362	0.720, 2.576	1.248	0.640, 2.434	19.6%	1.254	0.666, 2.359
**Education level**							
Degree and Higher degree (Ref)	1	–	1	–	9.3%	1	–
A Level or equiv	1.532	1.197, 1.962	1.488	1.124, 1.968	13.3%	1.480	1.126, 1.946
GCSE or equiv	2.153	1.664, 2.784	2.676	2.043, 3.506	21.6%	2.520	1.918, 3.312
Other Qualification	1.395	0.906, 2.146	2.464	1.588, 3.824	20.3%	2.109	1.399, 3.178
None	1.484	0.893, 2.466	3.362	1.950, 5.794	25.7%	3.539	2.058, 6.085

Dependent Variable: unlikely or very unlikely binary outcome (reference category = likely or very likely).

^1^Including age (5-year age bands), gender, ethnicity, country of birth, UK country of residence, education level and shielding status.

^2^Based on the reference group males aged 45–54 years.

Higher vaccine hesitancy was seen in most minority ethnic groups compared to the White British or Irish group. The highest odds were seen in the Black or Black British group (OR 13.42, 95% CI:6.86, 26.24) and the Pakistani or Bangladeshi groups (OR 2.54, 95% CI:1.19, 5.44), and adjustment for covariates made relatively little difference to these associations. Participants who were not born in the UK did not have greater odds of vaccine hesitancy (OR 0.99 95% CI: 0.67, 1.48) in model 2. Furthermore, in model 2, there were no substantive differences in vaccine hesitancy by country of residence.

The risk of vaccine hesitancy was inversely related to education in model 2. Compared to those with degree-level education there were raised odds of vaccine hesitancy among those with A-Level or equivalent qualifications (OR 1.48, 95% CI:1.13, 1.95), GCSE or equivalent (OR 2.52, 95% CI:1.92, 3.31), and no educational qualifications (OR 3.54, 95% CI:2.06, 6.09), indicating a socioeconomic gradient in vaccine hesitancy.

## 4 Discussion

The vast majority (82%) of UK adults are willing to take up a COVID-19 vaccine if offered with 18% being vaccine hesitant however marked differences exist across population subgroups. Older age and being male are strongest drivers of the risk of COVID-19 death, they are less likely to be vaccine hesitant, suggesting the vaccination programme could yield large health benefits within the UK. Very large differences in vaccine hesitancy exist by ethnicity, with Black or Black British and Pakistani or Bangladeshi ethnic groups being most hesitant. However, not all minority ethnic groups had higher vaccine hesitancy, highlighting the importance of understanding heterogeneity between minority ethnic groups. Overall, the main reasons given for vaccine hesitancy were fears around side effects and future adverse effects of a COVID-19 vaccine. The main reasons for intended vaccine uptake relate to the avoidance of catching the virus or becoming very ill from it, but also to allow social and family life to return to normal. Vaccine efficacy and safety were identified as factors that would encourage vaccine uptake.

Our study has several strengths. To our knowledge, it is the first large representative study in the UK to survey participants on likely vaccine uptake or hesitancy and the reasons why a COVID-19 vaccine would be accepted or refused. It also did so at a time when new vaccine development and vaccine efficacy was highly reported in the media (end November 2020, but just before the first vaccine was approved on 2nd December 2020). The four countries of the UK are now rolling out a vaccination programme commencing with those most at risk of mortality from COVID-19. The findings reported here, provide evidence on the groups who need targeting and arguments that may be most persuasive for them.

There are also weaknesses of our study which should be noted. The survey is web-based so non-participation may have introduced bias into the results. However, and following recommended practice for this dataset, the results were weighted to account for non-response and attrition (Benzeval et al., 2021). Furthermore, our main pattern of findings of which ethnic, age and gender groups were most likely to be vaccine hesitant was also seen in unweighted analyses (results not shown). Secondly, small numbers did not allow for detailed analysis of some ethnic groups. Additionally, we did not ask about the different types of vaccinations being developed and whether this would have any bearing on vaccine hesitancy. While we highlight associations between vaccine hesitancy and range of socio-demographic factors, the purpose of this analysis was descriptive. Willingness to be vaccinated is influenced by public health and other communications, as well as a broader range of social factors. These associations should therefore not be interpreted as immutable effects but rather guide vaccination planning.

There have been some other smaller UK studies of COVID-19 vaccine hesitancy which have not been based on representative samples. These studies indicated that 14% of participants were unwilling to receive a COVID-19 vaccine with a further 22% being unsure as to whether they would take this, with only 64% willing to take a COVID-19 vaccine. (Paul et al., 2021) A study conducted in Ireland and UK during the early phase of the pandemic found vaccine hesitancy of 35% and 31% in these populations respectively (Murphy et al., 2021). A non-probability online survey in Autumn 2020 found 71.7% were willing to be vaccinated, 16.6% were very unsure, and 11.7% were very vaccine hesitant (Freeman et al., 2020). A Scottish survey found uptake figures to be slightly higher at between 74 and 78%. A poll by Ipsos MORI in late October 2020 found 67% of the UK public said they were very (42%) or fairly (25%) likely to take a COVID-19 vaccine (Ipsos, 2020). Our study suggests slightly higher vaccine uptake in the general UK population of 82% with vaccine hesitancy at 18%.

Given the age related focus of the Phase One vaccination roll out in the UK (Joint Committee on Vaccination and Immunisation, 2020), our study suggests that uptake is likely to be high in the target groups. However, the finding of greater vaccine hesitancy amongst some, but not all, ethnic minority groups is concerning and aligns with emerging evidence from other countries. This is not inevitable – studies focused on intention to receive vaccines prior to this pandemic have not consistently found greater levels of vaccine hesitancy among ethnic minority groups (Bish et al., 2020, Han et al., 2016). Nevertheless, it needs to be a key focus of the design of vaccination programmes, given the higher prevalence of COVID-19 among ethnic groups and the need to avoid exacerbating existing inequalities (Ethnicity subgroup of the Scientific Advisory Group on Emergencies, 2020). Our study also found that those who had lower education levels were more likely to be vaccine hesitant suggesting that there similarly needs to be added focus on increasing vaccine uptake with those experiencing socioeconomic disadvantage who are at more risk of COVID-19 (Niedzwiedz et al., 2020).

Vaccine hesitancy rates vary by country and population subgroup. However, international evidence across several studies suggests that approximately 25% of the general population are hesitant about accepting a COVID-19 vaccine (Peretti-Watel et al., 2020, Detoc et al., 2020, Grech et al., 2020, Dror et al., 2020, Ward et al., 1982, Wang et al., 2020). A systematic review of studies on willingness to be vaccinated suggests 60% of people intend to be vaccinated, indicating the UK may be better placed to utilise vaccination to address the pandemic (Robinson et al., 2020). In some countries, such as Italy, previous vaccination rates suggest uptake may be too low to stop the spread of COVID-19 (Palamenghi et al., 2020). Our study suggests that this is not the case in the UK and if everyone who has said they are likely or very likely to take up the vaccination if offered, actually get vaccinated, coverage in the UK could be high enough to achieve herd immunity. Existing research for vaccine hesitancy suggests that key drivers for uptake were perceived efficacy of a vaccine, concern over negative adverse effects and safety of a vaccine (Paul et al., 2021, Dror et al., 2020, Yeung et al., 2016). Our study also found fears over adverse effects and safety to be key reasons for vaccine hesitancy, especially future negative effects. However, our study also adds that knowing that a vaccine is effective in reducing the spread of COVID-19 could increase uptake.

## 5 Conclusion

Our study has important practical implications for public health policy. There are identifiable subgroups of the UK population who are more likely to be vaccine hesitant. Vaccine hesitancy is a complex problem (Shen, 2020) and as such a range of practical steps need to be undertaken to increase uptake. Firstly, the subgroups we have identified as being vaccine hesitant should be included in the planning and development of any engagement programmes. There is the potential to widen health inequalities without deliberate efforts to engage those groups who are most likely to be affected by COVID-19 and least likely to take up a vaccine. Initiatives to improve uptake in Black ethnic groups within the UK should be an urgent priority – for example, by working in close partnership with communities and making use of community champions (Woodall et al., 2013). While universal and targeted educational interventions are necessary to enable the public to understand the importance of vaccination and are ethically and politically acceptable, they are not enough to modify behaviour or increase confidence (Verger and Dube, 2020). Full endorsement from regulatory bodies is likely to increase confidence (Kreps et al., 2020), but efforts to combat misinformation, especially around vaccine safety, may be warranted. The rise in vaccine hesitancy as a result of misinformation about safety coincides with the rise in social media, a growing platform for the anti-vaccination movement (Shen, 2020, Verger and Dube, 2020). A concerted effort to engage with younger adults both online and through traditional communication channels will be needed if confidence in a vaccine is to be achieved and vaccine uptake is to improve in this group, subject to them being included in future vaccination rollout.

Further detailed qualitative research should investigate the reasons for vaccine hesitancy with the subgroups identified as highly hesitant and approaches to overcoming them. While compulsory vaccination is unlikely in the UK, we have not asked about the acceptability of mandated vaccination for certain situations, e.g. immunisation passports or restrictions based on vaccination status. Further research would be required to understand whether a form of mandating vaccination would be acceptable to the UK population, for example only allowing those vaccinated to visit care homes or travel restrictions based on vaccination status. As vaccination programmes continue to be implemented, ongoing monitoring of uptake and vaccination attitudes are needed.

## Appendix A

Vaccine hesitant respondents were asked to state the main reason for their decision. “What is the main reason you would not take the vaccine?”

The chart shows main vaccine hesitancy reason by ethnic group

*Please note: Where crosstabulation created cells of values < 4, categories were combined.*
